# Efficacy and safety of second-line therapy by S-1 combined with sintilimab and anlotinib in pancreatic cancer patients with liver metastasis: a single-arm, phase II clinical trial

**DOI:** 10.3389/fimmu.2024.1210859

**Published:** 2024-02-01

**Authors:** Xin Qiu, Changchang Lu, Huizi Sha, Yahui Zhu, Weiwei Kong, Fan Tong, Qiaoli Wang, Fanyan Meng, Baorui Liu, Juan Du

**Affiliations:** ^1^ The Comprehensive Cancer Center of Drum Tower Hospital, Medical School of Nanjing University & Clinical Cancer Institute of Nanjing University, Nanjing, China; ^2^ The Comprehensive Cancer Center of Drum Tower Hospital, Clinical College of Traditional Chinese and Western Medicine, Nanjing University of Chinese Medicine, Nanjing, China

**Keywords:** advanced pancreatic cancer, liver metastasis, S-1, immunotherapy, antiangiogenic therapy

## Abstract

**Background:**

Pancreatic adenocarcinoma carries a grim prognosis, and there are few recognized effective second-line treatment strategies. We attempted to evaluate the efficacy and safety of a combination of S-1, sintilimab, and anlotinib as a second-line treatment in pancreatic cancer patients with liver metastasis.

**Methods:**

Pancreatic cancer patients with liver metastases were recruited. S-1 was administered orally at 25 mg/m^2^ bid, anlotinib was administered orally at 12 mg qd from day 1 to day 14, and sintilimab was administered intravenously at 200 mg on day 1. This method was repeated every 21 days, and the therapeutic effect was evaluated every 3 cycles. The primary outcome was the objective response rate (ORR).

**Results:**

Overall, 23 patients were enrolled in this study of whom 19 patients had objective efficacy evaluation. The ORR was 10.5% (95% CI 0.4%–25.7%) in the evaluable population. The progression-free survival (PFS) was 3.53 (95% CI 2.50–7.50) months, and the overall survival (mOS) was 8.53 (95% CI 4.97–14.20) months. Grade 3 adverse events were 26.1%, and no grade 4 or above adverse events occurred. High-throughput sequencing was performed on the tumor tissues of 16 patients; patients with HRD-H (n = 10) had shorter PFS than those with HRD-L (n = 6) (2.43 vs. 5.45 months; *P* = 0.043), but there was no significant difference in OS between the two groups (4.43 vs. 9.35 months; *P* = 0.11).

**Conclusions:**

This study suggests the advantage of S-1 combined with sintilimab and anlotinib in extending OS as a second-line therapy in pancreatic cancer patients with liver metastasis.

**Clinical Trial Registration**: ChiCTR2000030659

## Introduction

Pancreatic ductal adenocarcinoma (PDAC) is one of the worst digestive tract malignancies, with an average 5-year survival rate of 9% at most (1). The rates of morbidity and mortality of pancreatic cancer are increasing every year, and it is projected to become the second leading cause of cancer-related deaths in the USA before 2030 (2). 85% of patients were unable to undergo radical surgery at the time of first diagnosis, and the 5-year survival rate was less than 30% for patients who received radical resection (R0 resection status) (3, 4). Currently, there remain few standard second-line treatments for advanced pancreatic cancer. Results from the phase III study of NAPOLI-1 demonstrated that combination chemotherapy arms have been reported to show considerable efficacy in the second-line treatment for advanced pancreatic cancer patients (5). However, the incidence of grade 3/4 adverse reactions in the combination chemotherapy group was higher, and the physical condition of the second-line treatment patients with advanced pancreatic cancer is so poor that they may not be able to tolerate the combined chemotherapy regimen; compared with traditional chemotherapies, adverse events of anti-angiogenic therapy and immunotherapy are different. Therefore, this study was carried out to explore an effective and tolerable second-line treatment of metastatic pancreatic cancer.

In general, most guidelines recommend the use of 5-FU-based therapy for patients with advanced pancreatic cancer after failure of gemcitabine-based therapy. S-1 monotherapy in gemcitabine-refractory metastatic pancreatic cancer has been reported to show some efficacy in a previous phase II trial, the objective response rate (ORR) was 15%, overall survival (OS) was 4.5 months, and most of those adverse reactions were tolerable (6). Although there has been no evidence of phase III clinical studies, S-1 could play an important role in the clinical treatment of advanced pancreatic cancer.

In recent years, the anti-cytotoxic T-lymphocyte-associated protein 4 (anti-CTLA-4) and against programmed cell death receptor 1 (PD-1) and its ligand (PD-L1) have gradually become the focus of research and development and have fully proven their potential as “cancer killers”. Immune checkpoint inhibitors (ICIs) have achieved full prospects in advanced non-small cell lung cancer (7) and melanoma (8), and ICIs have achieved excellent results in the treatment of various tumors, especially in patients with programmed cell death-ligand 1 (PD-L1) positive, microsatellite instability-high/deficient mismatch repair (MSI-H/dMMR), or tumor mutational burden-high (TMB-H) (9–11). Unfortunately, only a minority of patients with pancreatic cancer meet these conditions and limited clinical activity of ICIs was observed. Furthermore, recent studies have reported that homologous recombination deficiency (HRD) can predict the therapeutic outcomes of immunotherapy (12).

The antitumor effect of antiangiogenic drugs have achieved excellent results in the treatment of various solid tumors. Unfortunately, only limited clinical activity of antiangiogenic drugs was observed in patients with pancreatic cancer. Chemotherapy plus antiangiogenic drugs as a first-line treatment for advanced pancreatic cancer did not improve patients OS compared with chemotherapy alone (13, 14), which is likely due to the profoundly suppressive tumor immune microenvironment (15, 16). Previous studies have shown that strategies combining anti-angiogenic therapy and immunotherapy seem to have the potential to tip the balance of the tumor microenvironment and improve treatment response in the treatment of primary liver cancer (17, 18). Moreover, chemotherapy combined with antiangiogenic drugs and immunotherapy has achieved remarkable results in a variety of cancers (19–21). This combination therapeutic regime may lead to better outcomes for patients with pancreatic cancer liver metastasis.

## Methods

### Patients

**Figure 1:**
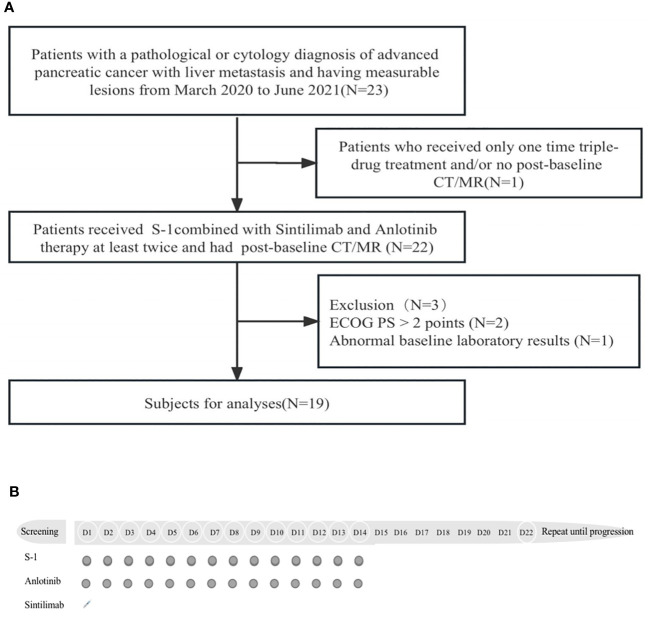
**(A)** Flow diagram of the study population. CT, computed tomography; MR, magnetic resonance; ECOG PS, Eastern Cooperative Oncology Group performance status. **(B)**, Protocol of drug administration.

This was a prospective study involving patients who received S-1 combined with sintilimab and anlotinib therapy for second-line treatment, from March 2020 to June 2021, at the Comprehensive Cancer Centre of Drum Tower Hospital, Clinical Cancer Institute of Nanjing University. To determine the sample size for this clinical trial, we hypothesized and estimated ORR improvement of standard second-line therapy for pancreatic cancer. Inclusion criteria were as follows: (1) histological or cytological diagnosis of pancreatic cancer with liver metastasis and having measurable lesions refer to NCCN guidelines; (2) an Eastern Cooperative Oncology Group performance status (ECOG PS) is ≤2 points; (3) received at least two cycles of the combination therapy and had a post-baseline computed tomography scan; (4) adequate bone marrow function, defined as platelets greater than 100 × 10^9^/L and/or WBC greater than 3.5 × 10^9^/L. Adequate liver functions was defined as ALT and AST less than the 5× upper limit of normal. Adequate renal function was defined as creatinine clearance upper than 30 mL/min. Patients with any of the following criteria were excluded: Prior or concurrent other malignancy and/or hypersensitivity to study drugs, and patients over the age of 80 years or any severe concomitant disease included autoimmune disease. Flow diagram of the study population is shown in **Figure 1A**.

### Treatment

All patients who enrolled in the trial received S-1 and anti-PD-1 and anti-angiogenic drugs in 21-day cycles. S-1 was administered orally at 25 mg/m^2^ bid from day 1 to day 14, sintilimab was administered intravenously at 200 mg on day 1, and anlotinib was administered orally at 12 mg qd from day 1 to day 14. This method was repeated every 21 days until disease progression. **Figure 1B** shows the drug administration protocol of the regimen. The therapy response was evaluated with CT or MR every 3 cycles. During the treatment period, concomitant medications for other conditions were recorded (including auxiliary drug treatment). The criteria for exiting the trial were that the patient’s imaging suggested disease progression or occurrence of unacceptable toxicity. Toxicity was monitored during combination therapy.

### Assessment

The baseline assessments were performed by laboratory samples and imaging tests before the first treatment. Prior to each cycle of the combination therapy, physical examination and laboratory examination were performed, including blood routine, liver and kidney function, and carbohydrate antigen 19-9 (CA19-9). According to the Response Evaluation Criteria in Solid Tumors (version 1.1), the treatment response was classified into four grades: complete response (CR), partial response (PR), stable disease (SD), and disease progression (PD). The safety of treatment was observed according to the National Cancer Institute Common Terminology Criteria for Adverse Events (version 4.0). All patients were followed up until the date of death, or the last follow-up date (no later than 01/10/2021). The primary endpoint was ORR as determined by RECISTv1.1, which refers to the proportion of patients whose tumors shrink by a certain amount and remain for a certain period of time. The secondary endpoints were disease control rate (DCR), OS and progression-free survival (PFS), and adverse effects. DCR was defined as the proportion of patients with complete responses, partial responses, and stable disease according to RECISTv1.1. OS was defined as the duration from the beginning of this therapy to death or last follow-up from any cause. Moreover, PFS was defined as the time from the beginning of treatment to disease progression or death. NGS analysis was carried out at OrigiMed (Shanghai, China), a College of American Pathologists-accredited and Clinical Laboratory Improvement Amendments-certified laboratory, using a 450-gene comprehensive assay. At least 50 ng of DNA was extracted from each 40-mm formalin-fixed paraffin-embedded (FFPE) tumor sample using a DNA Extraction Kit (QIAamp DNA FFPE Tissue Kit) in accordance with the manufacturer’s protocols. This panel encompassed all coding exons of 450 cancer-related genes and 64 selected introns of 39 genes that are frequently rearranged in solid tumors. Furthermore, the probe density was increased to ensure high capture efficiency in the conservatively low-read-depth regions. Peripheral blood was sampled from each patient as the normal control sample for genomic profiling. The genes were captured and sequenced with a mean coverage of 900× for FFPE samples and 300× for matched blood samples using an Illumina NextSeq 500 Platform (Illumina Incorporated, San Diego, CA, USA). There were 16 patients who had samples available for HRD measures, and we assessed the HRD score by the SNP array with genomic scar scanning. Scanning of genomic scar was performed using the scarHRD R package, which estimates the level of the three HR deficiency measures using NGS data and has been described previously (22). In this study, a high HRD score was defined as 35 or greater (23).

### Statistical methods

All analyses were performed among the participants. The data were also analyzed using version 4.0.1 (R Foundation) and SPSS software (version 24.0). Categorical variables were summarized by descriptive statistics with the 95% confidence interval Wilson score (CIs). Continuous variables were expressed as median (range). OS and PFS were performed using the Kaplan–Meier method, and the significance of the difference in estimates of median survival with two-sided 95% CIs was calculated using the log-rank test. *P* < 0.05 was considered statistically significant.

## Results

### Study population

There were 23 patients enrolled in this study, of whom 19 were evaluable [one patient was excluded due to not having had a post-baseline computed tomography scan; exclusions were made because of ECOG PS > 2 points (N = 2) and abnormal baseline laboratory results (N = 1)], 12 (47.3%) male patients, and 11 (52.6%) female patients with a median age of 61 years (range of 39–78 years). The ECOG performance status of all patients was 1–2. All subjects had received first-line chemotherapy with a Nab-paclitaxel and gemcitabine regimen. Four patients (17.4%) had previously received pancreatic radiotherapy. In total, all pancreatic cancer patients had liver metastases, five of which (21.7%) had more than two organ metastases, and lung metastasis and peritoneal metastasis were the most common. The baseline characteristics of the patients are summarized in **Table 1**.

**Table 1:** The eligible cohort of patients for the purposes of the present analysis.

Characteristic	Present study cohort (N=23)	No.(%)
Age (years)		
Median (range)	61(39-78)	
Sex		
Male	12	52.2%
Female	11	47.8%
ECOG PS		
0 or 1	17	73.9%
2	6	26.0%
Tumor size (mm)	26(11-89)	
CA 19-9 level at baseline		
median (range) , u/ml	6657(54.7-119100)	
TMB		
TMB-L(TMB<10 Muts/Mb)	15	65.2%
TMB-H(TMB≥10 Muts/Mb)	1	4.3%
NA	7	30.4%
MSI		
MSI-H	0	0%
MSS	16	69.6%
NA	7	30.4%
History of Resection	4	17.4%
History of radiation therapy	4	17.4%
Pancreatic tumor location		
Head	7	30.4%
Body	12	52.2%
Tail	4	17.4%
Metastatic		
Liver	23	100%
Lung	5	21.7%
Peritoneum	6	26.1%
Bone	1	4.3%
No.of metastatic sites		
≤2	18	78.2%
>2	5	21.7%

ECOG PS, Eastern Cooperative Oncology Group performance status, CA19-9, carbohydrate antigen 19-9, TMB, tumor mutational burden, MSI, microsatellite instability.

### Response

**Figure 2:**
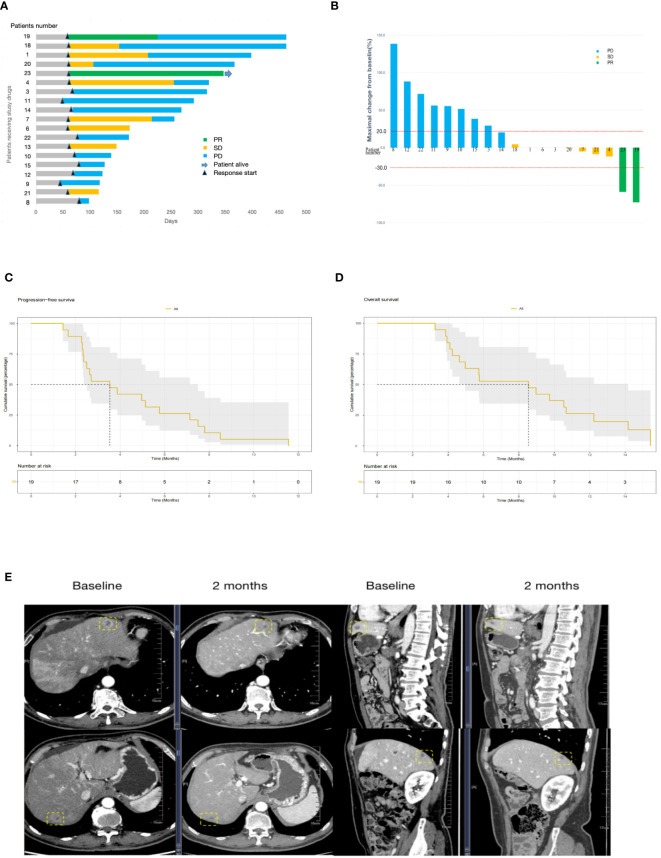
**(A)**, Swimmer plot analysis for evaluable patients (N = 19). The numbers 1 to 23 are patients’ numeral order. Blue, disease progression; yellow, stable disease; green, partial response. Survival is measured in days. **(B)**, Waterfall plot of best change of tumor size from baseline (N=19). The numbers 1 to 23 are patients’ numeral order. Blue, disease progression; yellow, stable disease; green, partial response. Survival is measured in days. PFS **(C)** and OS **(D)** in the 19 patients with advanced pancreatic cancer who received S-1 combined with sintilimab and anlotinib. **(E)** Computed tomography images of the liver metastasis of patient no. 23. A partial response was achieved after 3 cycles of treatment with the sum of the largest diameters of liver metastases, which decreased from 6.6 cm to 2.7 cm from baseline according to RECISTv1.1. PFS, progression-free survival, OS, overall survival.

Altogether, 23 patients were enrolled and 19 patients received more than two treatment cycles and the objective efficacy was evaluated. Response by RECISTv1.1 in the evaluable population included two patients (10.5%) who had a confirmed partial response and eight patients (42.1%) who were stable. However, there was no complete response. The ORR was 10.5% (95% CI 0.4%–25.7%), and the DCR was 52.6% (95% CI 27.9%–77.4%) in the study (**Table 2**). The best changes compared with the baseline tumor size and the overall treatment results are presented using swimmer charts, shown in **Figures 2A, B**.

**Table 2:** Response of the evaluable population.

	Cases	%
Rate of objective response		10.5%
Rate of disease control		52.6%
Response		
Complete response	0	
Partial response	2	10.5%
Stable disease	8	42.1%
Progressive disease	9	47.4%

The potential of S-1 combined with sintilimab and anlotinib for pancreatic cancer patients with liver metastasis was evidenced in patient no. 23 who is a 63-year-old man. He was treated with gemcitabine plus Nab-paclitaxel in the first-line therapy for 9 months. The number of hepatic metastases increased, and the efficacy was assessed as disease progression. At the beginning of the second-line therapy, there were three measurable liver metastasis lesions on computed tomography (CT). After 3 cycles of treatment, CT images showed that the lesions had shrunk to very good partial remission, with 59% tumor burden reduction from baseline (**Figure 2E**).

### Survival and subgroup analyses

**Figure 3:**
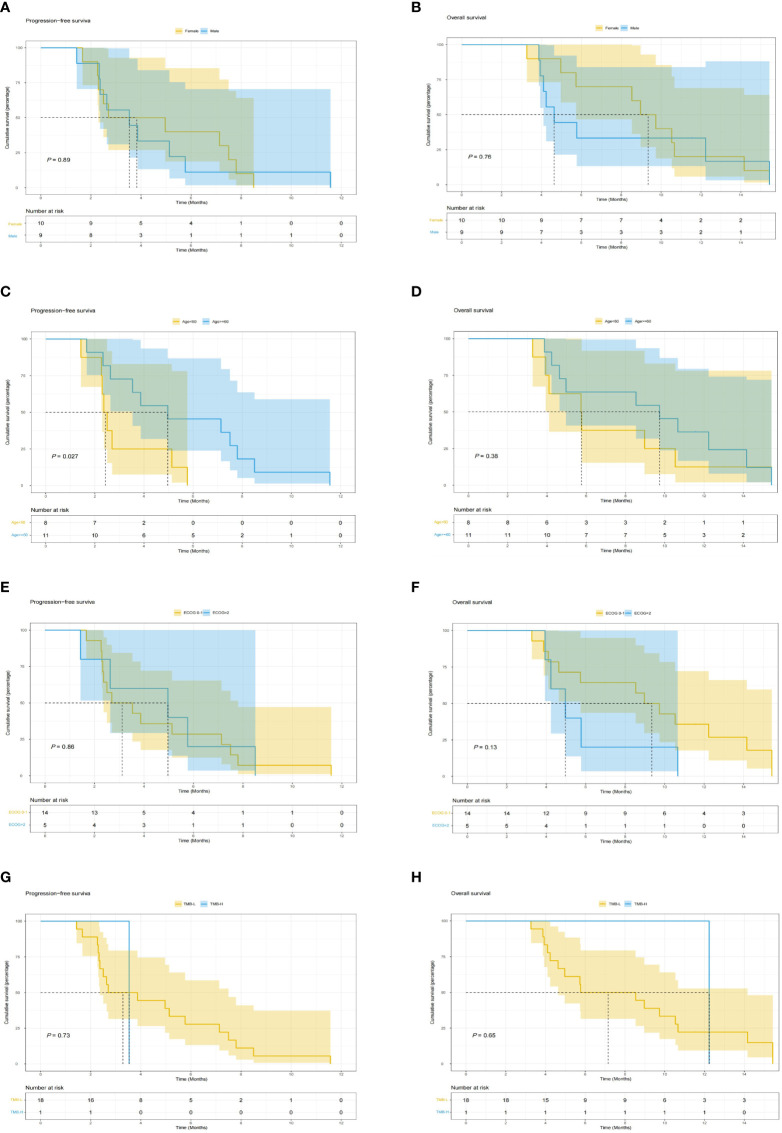
Kaplan–Meier analysis of survival The Kaplan–Meier curves of **(A)** PFS and **(B)** OS stratified by sex. The Kaplan–Meier curves of **(C)** PFS and **(D)** OS stratified by age. The Kaplan–Meier curves of **(E)** PFS and **(F)** OS stratified by ECOG PS. The Kaplan–Meier curves of **(G)** PFS and **(H)** OS stratified by TMB. PFS, progression-free survival, OS, overall survival, ECOG PS, Eastern Cooperative Oncology Group performance status, TMB, tumor mutational burden.

The last follow-up date was 15/05/2022. In the final analysis, 18 patients (95%) died and only one patient survived. The median PFS and OS were 3.53 (95% CI 2.50–7.50) months and 8.53 (95% CI 4.97–14.20) months, respectively (**Figures 2C, D**). The OS of female patients treated with S-1 combined with sintilimab and anlotinib as pancreatic cancer second-line therapy was 9.35 months, whereas that of male patients was 4.63 months, but *P* > 0.05 (**Figure 3B**). Patients younger than 60 years have poorer mPFS compared with patients older than 60 years (mPFS 2.43 vs. 4.97 months, *P* < 0.05) (**Figure 3C**). In addition, ECGO PS > 2 points do not predict the worse outcome (**Figures 3E, F**). In only one patient (4.3%) with TMB-H, we failed to verify if the TMB status affects the responses to triple-drug treatment (**Figures 3G, H**).

### Adverse events

The intensity of most adverse events in the trial was grades 1–2. The most common hematological toxicities included leucopenia, neutropenia, and thrombocytopenia. The most common non-hematologic toxicities were fatigue (7/23) and nausea/emesis (5/23). The most common immunotherapy-related adverse events were hypothyroidism (3/23) and hyperthyroidism (3/23). One patient stopped taking anlotinib because of intolerable toxic side reactions of hypertension. Grade 3 adverse events occurred in 26.1% of patients, and no grade 4 or higher adverse events or treatment-related deaths were observed (**Table 3**).

**Table 3:** Adverse event in this trial.

TRAEs	Grade		
	1-2	3	≥4
	N (%)	N (%)	N (%)
Leucopenia	9 (39.1%)	2 (8.7%)	0 (0%)
Anemia	5 (21.7%)	1 (4.3%)	0 (0%)
Thrombocytopenia	10 (43.5%)	1 (4.3%)	(0%)
Increased ALT or AST	0 (0%)	0 (0%)	0 (0%)
Fatigue	7 (30.4%)	0 (0%)	0 (0%)
Nausea or emesis	5 (21.7%)	0 (0%)	0 (0%)
Colitisor Diarrhea	1 (4.3%)	0 (0%)	0 (0%)
Hypertension	2 (8.7%)	0 (0%)	0 (0%)
Mucositis oral	0 (0%)	1 (4.3%)	0 (0%)
Skin hyperpigmentation	3 (13.0%)	0 (0%)	0 (0%)
irAEs			
Rash	0 (0%)	0 (0%)	0 (0%)
Pneumonitis	0 (0%)	0 (0%)	0 (0%)
Hypothyroidism	3 (13.0%)	0 (0%)	0 (0%)
Hyperthyroidism	3 (13.0%)	0 (0%)	0 (0%)
Myositis	0 (0%)	1 (4.3%)	0 (0%)
Hepatitis	0 (0%)	0 (0%)	0 (0%)
Adverse event leading to death	0 (0%)	0 (0%)	0 (0%)

TRAEs, treatment-related adverse events; irAEs, immune-related adverse events; ALT, alanine aminotransferase; AST, aspartate aminotransferase.

### Genetic mutation status

The tumor tissues of 16 patients underwent high-throughput genome sequencing, and six patients were evaluated as HRD-High and 10 patients were evaluated as HRD-Low. Patients with HRD-H had shorter PFS than those with HRD-L (2.43 months vs. 5.45 months; *P* = 0.043). Patients with HRD-H had shorter OS than those with HRD-L; however, there was no significant difference in OS (4.43 months vs.9.35 months; *P* = 0.11) (**Supplementary Figure**). In addition, we found that BRAF, PRSS1, PANBP2, RUNX1T1, and other genetic mutations were associated with efficacy of patients who receive combination treatment, but because of the small sample size, it was not possible to determine the significance of the mutant state in pancreatic cancer treatment (**Table 4**).

**Table 4:** Radiographic,change of tumor size and molecular response characteristics.

Patient number	Radiographic respones	Best change of tumor size	Number of gene mutations	Mutation state	Tumor mutation burden
1	SD	0%	4	KRAS G12D 12%ERBB4 2% KMT2C 14% SMAD4 6%	MSS; TMB-L
3	PD	30%	14	KRAS G12V 34% SERPINB3 c.223-1G>A 25% PIK3CG E602D 14%	MSS; TMB-L
4	SD	-12%	4	NTRK,BRAF	MSS; TMB-L
6	SD	0%	3	KRAS G12V 22% TP53 V216M 19% TRIO c.8019+6T>C 17%	MSS; TMB-L
7	SD	-5%	3	Gene amplification CALR;H3-3A;SGK1	MSS; TMB-L
8	PD	139%	Indeterminable	Indeterminable	MSS; TMB-L
9	PD	56%	6	ARID1A R1461* 27% BRAF G469S 37% MYCN D343V 19% RANBP2 c.2202+2T>G 20% RUNX1T1 G23Rfs*4 15% TP53 R196* 27%	MSS; TMB-L
10	PD	52%	3	TP53 1%;KRAS 1% ;SMAD4 1%	MSS; TMB-L
11	PD	56%	1	FFPE 1%	MSS; TMB-L
12	PD	88%	4	KRAS 38%;SMARCE1 ;BRD4;TP53 7%	MSS; TMB-L
13	SD	0%	Indeterminable	Indeterminable	Indeterminable
14	PD	20%	3	LRP1B 2%,KRAS 1% ;TP53 1%	MSS; TMB-L
15	PD	38%	1	ERBB2(HER2)7%	MSS; TMB-L
18	SD	4.4%	3	FGFR4 G388R 40%;NSD2 E1099K 16% ;KRAS G12R13%	MSS; TMB-L
19	PR	-73%	7	KRAS G12V 39%;TP53 S166* 28%;GNA11 R213Q 21%;CTNNA2 A673V 21%;ACVR2A R57W 20%	MSS; TMB-L
20	SD	-2.2%	48	CARD11 25%;EGFR 31%;···	MSS;TMB-H 37.4(Muts/Mb)
21	SD	-9%	Indeterminable	Indeterminable	Indeterminable
22	PD	72%	5	CDKN2A A86D 4% KRAS G12R 14% PIK3C2B S242L 4% ROS1 A1423G 8% TP53 R282W 12%	MSS; TMB-L
23	PR	-59%	Indeterminable	Indeterminable	Indeterminable

TMB, tumor mutational burden, MSI, microsatellite instability.

## Discussion

Pancreatic cancer is a malignant disease with poor prognosis. Up to now, there are limited options for standard second-line treatment of advanced pancreatic cancer. The lack of early diagnosis and low response to treatments of pancreatic cancer is mainly attributed to its specific anatomical characteristics, highly aggressive histology, and tumor microenvironment. Since patients of advanced pancreatic cancer with liver metastases are usually in poor physical condition and accompanied by many other accompanying symptoms, most patients may not be able to tolerate combination chemotherapy at second-line therapy. There is also no clear evidence that fluoropyrimidine (5-fluorouracil or capecitabine), gemcitabine, oxaliplatin, irinotecan, or a combination of oxaliplatin and irinotecan can improve patient survival after the failure of first-line chemotherapy.

Therefore, the choice of second-line treatment for pancreatic cancer is more difficult. In the phase III study of NAPOLI-1, the OS of combination chemotherapy arms was 6.1 months, and it was 1.9 months longer than that of the 5-FU/LV group (5). Modified FOLFIRINOX or S-1 monotherapy as second-line treatment for pancreatic cancer patients in a randomized controlled study has shown that combination chemotherapy increased OS than S-1 treatment alone. We can see that combination therapy has extended the duration of OS when compared with S-1 alone, but it comes at the expense of an increased toxic side effect (24). In this single-arm phase II clinical trial, two patients (10.5%) had confirmed partial response and eight patients (42.1%) obtained a stable disease; the median PFS and OS were 3.53 months and 8.53 months, respectively. Moreover, the efficacy and safety of triple drug treatment are acceptable.

Immunotherapy provides a promising method for the treatment of many gastrointestinal malignancies. In recent years, some small-sample clinical trials have achieved positive results with anti-PD-1 antibodies combined with chemotherapy drugs in advanced pancreatic cancer. In a phase II clinical trial, after initial treatment based on 5-FU or gemcitabine, the median OS of durvalumab monotherapy or durvalumab plus tremelimumab was 3.6 and 3.1 months, respectively (25). A phase II clinical trial was designed to evaluate the efficacy and safety of the CXCR4 antagonist BL-8040 combined with pembrolizumab and chemotherapy for second-line therapy of metastatic pancreatic cancer. The overall response rate was 32%, and the median duration of response was 7.8 months (26). Currently, clinical trials suggest that immunotherapy combinations may be suitable for the treatment of pancreatic cancer.

In addition, anti-PD-1 antibodies and anti-angiogenesis drugs can be used in combination at primary liver cancer and other solid tumors to obtain good efficacy. Recently, an open-label phase III clinical trial has confirmed the efficacy of atezolizumab combined with bevacizumab for patients of unresectable hepatocellular carcinoma who had not previously received systemic treatment (17). Tumor invasion and metastasis through a constant crosstalk with the surrounding microenvironment are well known, and emerging evidence indicates that within the tumor microenvironment, abnormal tumor blood vessels foster immune-suppressive cell evasion, which in turn promotes tumor angiogenesis (15, 16, 27). This vicious circle leads to the ineffectiveness of single immunotherapy or antiangiogenic monotherapy. Accordingly, strategies combining anti-angiogenic therapy and immunotherapy seem to have the potential to tip the balance of the tumor microenvironment and improve treatment response (28). Furthermore, studies have previously shown that the ORR of pancreatic cancer patients with liver metastasis treated with antiangiogenic agents combined with immune checkpoint inhibitors was significantly higher than that of patients without liver metastasis (90.0% vs. 20.0%, *P* = 0.0017) (29). This study also demonstrated the efficacy of chemotherapy combined with immunotherapy and antiangiogenic agents in the second-line treatment of advanced pancreatic cancer patients with liver metastases, and the regimen is well tolerated.

HRD-H has been confirmed to be associated with homologous recombination repair and has also been reported to be associated with the therapeutic efficacy of platinum drugs and PARP inhibitors in solid tumors such as ovarian cancer (30) and pancreatic cancer (31). Some studies have reported that HRD can predict the therapeutic outcomes of immunotherapy and tumors with HRD-H usually have higher TMB (12, 32). However, other studies found that tumors with HRD-H have low TMB and HRD could not predict an enhanced benefit from immunotherapy (33, 34). In our study, we found that HRD-L was associated with better PFS of patients with pancreatic cancer receiving S-1 combined with sintilimab and anlotinib therapy, whereas the efficacy of this regimen was slightly worse for patients with HRD-H. Therefore, these results need to be further evaluated in large-sample clinical trials.

Nevertheless, there are several limitations in this study. First, this was a single-arm, phase II clinical trial with a small sample size, and while what we found was interesting, there was a lack of control groups and randomized trial results. Second, in this trial, one patient was diagnosed with suspected immune associated myocarditis after two cycles of treatment. For this patient, we stopped using sintilimab immediately and the symptoms were relieved gradually after high-dose glucocorticoid treatment. In previous reports, the incidence of immune myocarditis was approximately 0.06% (35). The incidence of immune myocarditis of sintilimab is also not common in previous reports. Therefore, immune-related adverse effects were considered to be manageable.

In conclusion, this phase II trial demonstrates the superiority of S-1 combined with sintilimab and anlotinib in extending OS in second-line therapy for gemcitabine-refractory pancreatic cancer patients with liver metastasis, which we will confirm in subsequent randomized trials. Furthermore, this triple-drug treatment has a lower incidence of high-grade adverse events.

## Data availability statement

The raw data supporting the conclusions of this article will be made available by the authors, without undue reservation.

## Ethics statement

The studies involving humans were approved by This clinical trial is approved by the ethics committee of Nanjing Drum Tower Hospital. The studies were conducted in accordance with the local legislation and institutional requirements. The participants provided their written informed consent to participate in this study. Written informed consent was obtained from the individual(s) for the publication of any potentially identifiable images or data included in this article.

## Author contributions

XQ and JD had full takes responsibility for the integrity of the data and the accuracy of the data analysis. Concept and design: BL and JD. Acquisition, analysis or interpretation of data: XQ, CL, HS, FT, QW, YZ, WK, FM. Drafting of the manuscript: XQ, CL, HS. Critical revision of the manuscript for important intellectual content: XQ, CL, HS, FT, QW, YZ, WK, FM, BL and JD. Supervision: BL and JD. All authors reviewed the manuscript. All authors contributed to the article and approved the submitted version.

## Supplementary material

Supplementary Figure 1 Progression-free survival **(A)** and overall survival **(B)** according to HRD score. (The cutoff points of HRD score is defined as 35 points.) Abbreviations: HRD-H, homologous recombination deficiency-high; HRD-L, homologous recombination deficiency-low.
