# Supplementary figures and images for: Efficacy and safety of second-line therapy by S-1 combined with sintilimab and anlotinib in pancreatic cancer patients with liver metastasis: a single-arm, phase II clinical trial
Source: Front Immunol. 2024 Feb 1;15:1210859. doi: 10.3389/fimmu.2024.1210859 (PMC10867188; doi:10.3389/fimmu.2024.1210859)

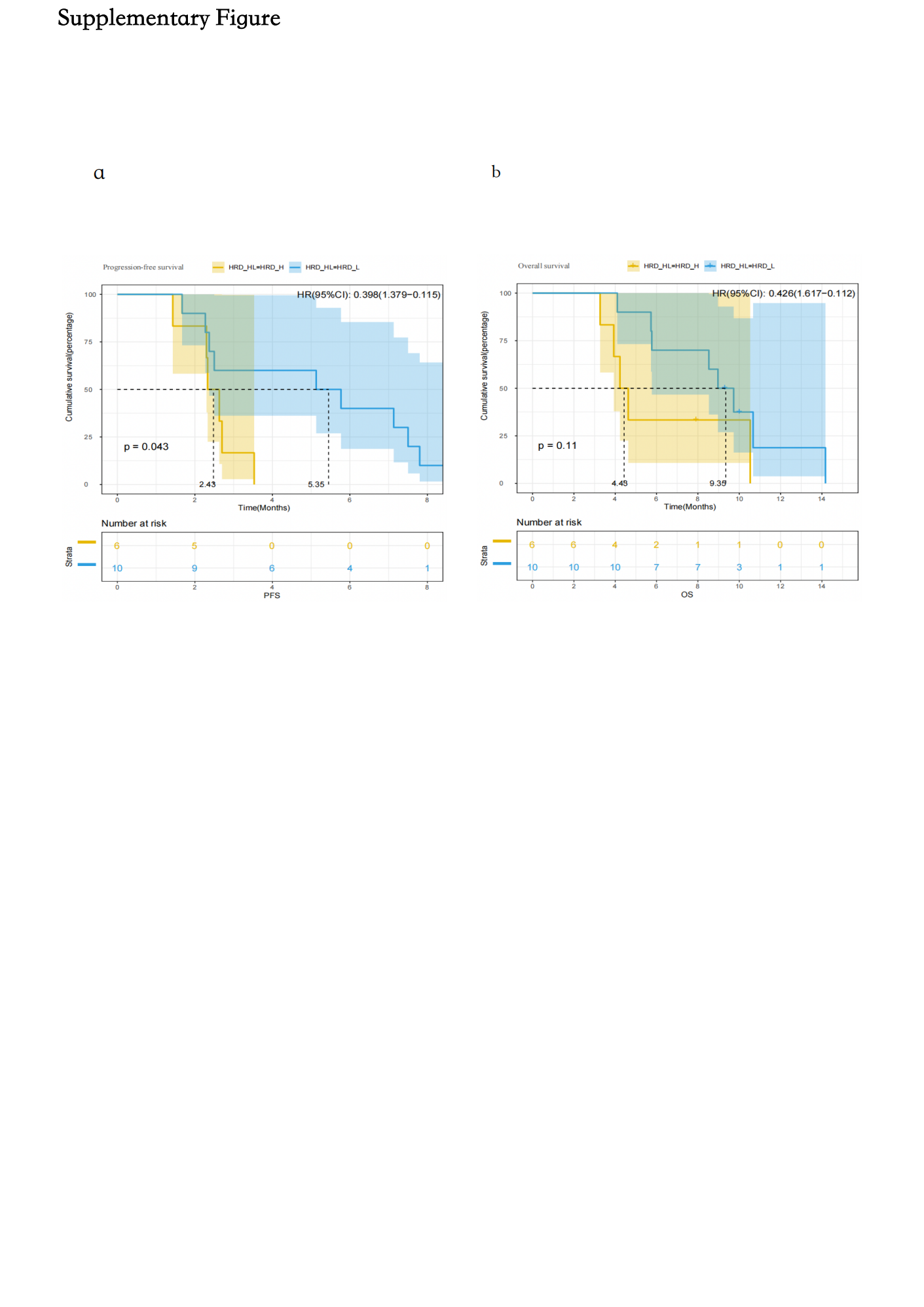

Supplement: Supplementary Figure 1 — Progression-free survival (A) and overall survival (B) according to HRD score. (The cutoff points of HRD score is defined as 35 points.) Abbreviations: HRD-H, homologous recombination deficiency-high; HRD-L, homologous recombination deficiency-low. [file Image_1.tif]
